# Modelling the Effects of MCM7 Variants, Somatic Mutations, and Clinical Features on Acute Myeloid Leukemia Susceptibility and Prognosis

**DOI:** 10.3390/jcm9010158

**Published:** 2020-01-08

**Authors:** Florin Tripon, Mihaela Iancu, Adrian Trifa, George Andrei Crauciuc, Alina Boglis, Delia Dima, Erzsebet Lazar, Claudia Bănescu

**Affiliations:** 1 Genetics Department, “George Emil Palade” University of Medicine, Pharmacy, Science and Technology of Târgu Mureș, 540139 Mureș, Romania; tripon.florin.2010@gmail.com (F.T.); andrei.crauciuc@gmail.com (G.A.C.); alinaboglis@gmail.com (A.B.); claudia.banescu@gmail.com (C.B.); 2 Genetics Laboratory, Center for Advanced Medical and Pharmaceutical Research of the “George Emil Palade” University of Medicine, Pharmacy, Science and Technology of Târgu Mureș, 540139 Mureș, Romania; 3 Department of Medical Informatics and Biostatistics, University of Medicine and Pharmacy “Iuliu Haţieganu” from Cluj Napoca, 400000 Cluj-Napoca, Romania; 4 Department of Medical Genetics, University of Medicine and Pharmacy “Iuliu Haţieganu” from Cluj-Napoca, 400000 Cluj-Napoca, Romania; 5 Department of Hematology, “Ion Chiricuta” Clinical Cancer Center, 400015 Cluj-Napoca, Romania; deli_dima@yahoo.com; 6 Department of Internal Medicine, “George Emil Palade” University of Medicine, Pharmacy, Science and Technology of Târgu Mureş, 540139 Mureș, Romania; erzsebetlazarbenedek@gmail.com

**Keywords:** *MCM7*, acute myeloid leukemia, *FLT3*, *NPM1*, *DNMT3A*, WBC, LDH

## Abstract

The main objective of the study was to evaluate the associations between *MCM7* rs2070215, rs1527423, and rs1534309 single nucleotide polymorphisms (SNPs) and acute myeloid leukemia (AML) risk and prognosis. The secondary objectives were to assess if any relationships existed between the mentioned SNPs and *FLT3*, *DNMT3A*, *NPM1* mutations with clinical outcomes and overall survival (OS) in AML patients. We investigated 281 AML cases and 405 healthy subjects. The results showed a significant association between a variant allele of rs2070215 (*p* = 0.007), CAT haplotype (*p* = 0.012), and AML susceptibility. No significant association was found between *MCM7* variant genotypes and overall survival of AML patients (*p* > 0.05), while several associations between somatic mutations, clinical and biological features, and poor OS were noticed. Lactate dehydrogenase (LDH) level ≥ 600 IU/L had a significant effect on the hazard of death (*p* = 0.004, HR = 1.49, 95% CI: 1.13–1.95). Our study showed that the variant allele of rs2070215, in the allelic model, and CAT haplotype were associated with AML susceptibility. The investigated *FLT3*, *DNMT3A*, and *NPM1* mutations were associated with the clinical and biological features and poor OS. LDH level ≥ 600 IU/L was associated with an increased hazard of death and this association remained significant when quantifying for effect modification by *FLT3* mutation status.

## 1. Introduction

Acute myeloid leukemia (AML) is characterized by a phenotypic and genetic heterogeneity. Several chromosomal rearrangements, somatic mutations, and epigenetic aberrations have been described to be implicated in the AML susceptibility, pathogenesis, prognosis, evolution, overall survival (OS), or in the treatment response of AML patients [1,2,3,4,5].

Recurrent structural and numerical chromosomal aberration and molecular markers, such as Fms like tyrosine kinase 3 (*FLT3*), Nucleophosmin 1 (*NPM1)*, and DNA methyltransferase 3 alpha (*DNMT3A*) mutations, were proved to have an impact on AML treatment response and prognosis [6,7,8,9,10,11]. High lactate dehydrogenase (LDH) value or hyperleukocytosis has been reported to be independent of poor prognostic factors in AML [12,13,14]. Associations between hyperleukocytosis and somatic mutations have also been reported [12]. The available biomarkers may explain the poor prognosis for just a part of all AML cases; therefore, it was supposed that other new markers might be involved [15], such as minichromosome maintenance complex component 7 (*MCM7)* gene variants, as they have been reported to be associated with relapse in pediatric and adult AML patients and their overall poor survival rate [9,15,16]. 

*MCM7* gene encodes an essential protein (Mcm7) of the mentioned complex with an important role in the control of cell cycle progression and initiation of DNA replication [15,16]. Mutations or single nucleotide polymorphisms (SNPs) that may affect DNA replication are a key step in the progression of many cancers [17,18]. DNA replication is an essential process for the survival; therefore, deficient DNA replication (or cell cycle) may affect the integrity of the genome (genomic instability is known to be a specific feature of cancer), becoming predisposed to different types of neoplasms, including hematological malignancies [19]. Thus, we may hypothesize that the *MCM7* gene may be a candidate marker involved in the development of various types of neoplasms [15]. 

Several studies have shown that overexpression of the *MCM7* gene is associated with the appearance of certain neoplasms such as gastric, hepatic, or esophageal cancer [20,21,22], non-Hodgkin’s lymphoma [23], acute myeloid leukemia [15,16], and chronic myeloid leukemia [17].

Overexpression of this gene was correlated (in the case of leukemia) with a negative prognosis, patients having multiple relapses, and a short overall survival [15,24], whereas suppression of *MCM7* gene expression was proposed as a new potential therapy for leukemia [17].

Different SNPs were reported to be associated with overexpression of the *MCM7* gene [25,26]. For example, the variant allele of rs2070215 increased the expression level of *MCM7* in T cell leukemia cell line and prostate cancer, resulting in increased cell proliferation and relapse of prostate cancer [15,25,26].

Previously, one study indicated that *MCM7* rs2070215, rs1527423, and rs1534309 SNPs might be used as prognostic markers for AML patients [15]. According to Ensembl genome browser (ensembl.org), rs2070215 consists of substitution of T with C, which leads to a missense variant, being a gain-of-function SNP, while rs1527423 and rs1534309 are intronic SNPs of the *MCM7* gene. No data were available regarding the association between *MCM7* SNPs and *FLT3*, *DNMT3A*, and *NPM1* mutations.

The main objective of this case-control study was to evaluate the relationship of SNPs of the *MCM7* gene, namely rs2070215, rs1527423, and rs1534309, with AML susceptibility and overall survival of AML patients. Subsequently, associations between the mentioned SNPs, somatic mutations such as *FLT3* ITD, *FLT3* D835, *DNMT3A* (codon R882), and *NPM1* (4-bp dup/ins, rs587776806, rs1554138188, rs1554128189), and clinical features of AML patients were evaluated. 

## 2. Materials and Methods

### 2.1. Patients and Controls

In this case-control study we included 686 adult subjects, comprising 281 patients with AML and 405 healthy controls. The mean age of AML patients at diagnosis was 55.11 ± 16.54 years (range values: 18–87 years), 47% (*n* = 132) being females and 53% (*n* = 149) males. In the control group, mean age was 55.98 ± 14.99 years (range values: 20–85 years), 50.6% (*n* = 205) being females and 49.4% (*n* = 200) males.

This study was approved by the Ethics Committee of the Clinical and Emergency Hospital of Târgu Mureş, Romania (10665/2019), and was performed in accordance with the Declaration of Helsinki in order to respect the ethical principles. All subjects included in this study, signed a written informed consent form.

### 2.2. Genotyping Investigation

DNA was extracted from leukocytes using the PureLink Genomic DNA kit (Thermo Fisher Scientific, Waltham, MA USA) according to the recommendations of the manufacturer. In cases with low white blood cell (WBC) count, blood quantity and protocol were adjusted. 

The mentioned SNPs were genotyped using TaqMan assays (Thermo Fisher Scientific, Waltham, MA, USA), namely C_118714, C_11987066, and C_8758823_10, and the 7500 Fast Dx Real-Time Polymerase Chain Reaction (PCR) system (Applied Biosystem). 

Somatic mutations analysis for *FLT3* ITD (internal tandem duplication), *FLT3* D835 (rs121909646, rs121913488, rs121913488, rs121913487, rs121913488, rs121913486), *DNMT3A* (codon R882, rs147001633 and rs377577594), and *NPM1* (4-bp dup/ins, rs587776806, rs1554138188, rs1554128189) were performed by PCR, restriction fragment length polymorphism PCR (RFLP-PCR), fragment analysis for *FLT3* ITD and *NPM*1, and randomly, capillary sequencing for *FLT3* D835 and *DNMT3A* confirmation, using the protocol previously reported [11]. 

Wild-type (reference) and variant alleles (alternatives) of mentioned SNPs and somatic mutations were indicated according to the Ensembl genome browser.

### 2.3. Statistical Analysis

Descriptive statistics as arithmetic mean ± standard deviation or absolute (number of cases) and relative frequencies (%) were used for describing genetic and clinical variables. The difference in distribution of the studied SNPs genotypes and alleles among AML patients and controls was tested using Chi-square (χ^2^). The bivariate associations between studied *MCM7* SNPs, somatic mutations (*FLT3*, *NPM1*, *DNMT3A*), combined somatic mutations (defined by “nonmutated”, “single-mutated”, and “double-/triple-mutated” status), and demographic or clinical variables were tested using the Chi-square (χ^2^) or Fisher exact test. In the case of a significant association between combined mutations in *FLT3*/*DNMT3A*, *FLT3*/*NPM*, and *NPM1*/*DNMT3A* or combined mutations in *FLT3/NPM1/DNMT3A* and clinical variables, a post hoc test using a pairwise Fisher’s exact test was performed, and adjusted *p*-values (pFDR) were reported in order to control false discovery rate (FDR).

Unconditional binomial logistic regression with main effects was used to assess the effect of investigated *MCM7* SNPs on the AML risk, using R software (version 3.6.0) [27]. We applied the Benjamini–Hochberg FDR procedure to the *p*-values of our family of genetic models (additive, dominant, and allelic) in order to account for multiple comparisons in the case of significant results.

The nonparametric Kaplan–Meier method with the Log-Rank test was performed for evaluating differences between survival rate for all potential predictors (somatic mutations, clinical factors). The Cox PH regression was used to assess the effects of *MCM7* variant genotypes and somatic mutations on survival time. The effect size was measured by crude Hazard ratios (HR) and adjusted HR for age group, gender, Eastern Cooperative Oncologic Group Scale (ECOG) performance status, cytogenetic risk group, type of AML, combined mutations (double- or triple-mutated versus one single- or nonmutated) in *FLT3/NPM1/DNMT3A*.

An estimated *p* < 0.05 was set as the level of statistical significance, and all statistical tests were two-sided. Linkage disequilibrium and Hardy–Weinberg equilibrium in AML cases and control groups were also determined by a “genetics” R package.

The haplo.stats R package (version 1.7.9) was used to test the association between statistically inferred haplotypes and AML risk [28,29]. The method implemented in this package assumed that haplotypes were ambiguous because of an unknown linkage phase of the genetic variants, and in addition, it could estimate the crude and adjusted magnitude of the effect of each haplotype on AML risk (crude OR and adjusted OR) with associated 95% confidence interval (CI).

## 3. Results

### 3.1. Description of AML and Control Groups

AML cases and control groups were similar in age (*p* = 0.484) and gender distribution (*p* = 0.348). According to European Leukemia Net (ELN) 2017 risk stratification, 55 (19.6%) of our patients were included in the low-risk category, 146 (52%) in the intermediate, 71 (25.3%) in the high-risk category, and for 9 (3.2%), we were unable to determinate the risk category. Regarding treatment and the response to therapy of our AML patients, we summarized that 122 (43.4%) were treated with low-dose (LD) therapy, 143 (50.9%) with high-dose (HD), and 16 (5.7%) with a combination of high-dose therapy and hematopoietic stem cell transplantation (HD + HSCT). The majority of them showed resistance to treatment (71, 25.3%) or did not respond and deceased a short time after diagnosis (69, 24.6%). On the other side, 43 cases (15.3%) had a complete remission (CR) without relapse, 49 (17.4%) had a partial remission (PR), and 49 (17.4%) relapsed after CR or PR.

### 3.2. MCM7 SNPs rs2070215, rs1527423, and rs1534309 and AML Risk

The genotype frequency for all three studied SNPs was consistent with Hardy–Weinberg equilibrium in AML cases and control groups (*p* > 0.05). The *MCM7* rs1534309 and rs1527423, rs2070215 and rs1527423, rs1534309 and rs2070215 SNPs were in linkage disequilibrium in both groups (*p* < 0.001).

There was no significant difference in genotype distribution for any of the three studied SNPs between AML patients and controls (Table 1). Regarding allelic model, we noticed that only variant C allele of rs2070215 had a significantly different distribution between groups (*p* = 0.007), observing a higher frequency of this allele in AML patients (197 cases, 32% vs. 207 controls, 25.6%). 

We also investigated the distribution of *MCM7* haplotypes between AML patients and controls (Table 2). Eight different haplotypes were obtained, of which 2 haplotypes with frequency <0.4% among AML patients or controls were not included in the estimation of AML risk because of their rarity. When haplotype 4 (GGT) was the considered reference, haplotype 1 (GAT) had a protective effect that remained significant after adjusting for conventional covariates like age and gender (OR = 0.71, 95% CI: 0.52–0.97). In the case of haplotype 6 (CAT), there was a significant positive association with AML risk, and it remained an independent risk factor after adjustment for age and gender of the patients (OR = 9.55, 95% CI: 1.13–80.40). 

### 3.3. MCM7 SNPs rs2070215, rs1527423, and rs1534309, Somatic Mutations (FLT3, NPM1, DNMT3A), and the Clinical Features of AML Patients

In Table 3 we illustrated the distribution of wild-type and variant genotypes of the investigated SNPs according to the clinical features of the patients. No significant association was observed between the genotypes and investigated features (such as WBC count, platelet (PLT) count, hemoglobin (Hgb), LDH level, blast percentage, AML subtypes, somatic mutations, treatment, and toxicity). In the case of rs2070215 and rs1527423 SNPs, distribution of variant genotypes differed between genders, the variant genotypes being more common in male AML cases (*p* = 0.026 and *p* = 0.05, respectively).

We also analyzed the association of the mentioned SNPs with blast percentage (defined as <70% vs. ≥70%, <60% vs. ≥60%, <50% vs. ≥50%), different ranges of WBC count (defined as <10,000 vs. ≥10,000, <15,000 vs. ≥15,000, <20,000 vs. ≥20,000 cells/mm^3^), and LDH level (defined as <600 UI/L vs. >600 UI/L), but the results were statistically insignificant (*p* > 0.05). Furthermore, we analyzed the association between SNPs and each of the somatic mutations (*p* > 0.05). The results were also statistically insignificant (*p* > 0.05) for the association between SNPs and combined somatic mutations. 

In addition, we performed an association analysis between *FLT3*, *NPM1*, and *DNMT3A* somatic gene mutations and LDH level, WBC count, and blast percentage. *FLT3* gene mutations were associated with high WBC count (>10,000 cells/mm^3^, *p* < 0.001), high LDH level (>600 IU/L, *p* = 0.001), and high bone marrow blast percentage (>70%, *p* < 0.001). *NPM1* gene mutation was associated with WBC count (>10,000 cells/mm^3^, *p* < 0.001) and high bone marrow blast percentage (>70%, *p* < 0.004), and *DNMT3A* gene mutation with WBC count (>15,000 cells/mm^3^, *p* = 0.019)

Furthermore, we examined the association of combined mutations in *FLT3*/*DNMT3A*, *FLT3*/*NPM*, and *NPM1*/*DNMT3A* and combined mutations in *FLT3*/*NPM1*/*DNMT3A* with WBC count, LDH level, and blast percentage. Significant results were obtained for all tested associations (*p* ≤ 0.002). The post hoc analysis (pairwise comparisons by Fisher’s exact test) revealed that the presence of somatic mutations in all three genes were significantly associated with high WBC (≥10,000 cells/mm^3^, *p*_FDR_ ≤ 0.005; ≥15,000 cells/mm^3^, *p*_FDR_ ≤ 0.001; ≥20,000 cells/mm^3^, *p*_FDR_ ≤ 0.001; ≥30,000 cells/mm^3^, *p*_FDR_ ≤ 0.015), with a high blast percentage (>70 %, *p*_FDR_ = 0.002), and high LDH level (≥600 IU/L, *p*_FDR_ < 0.001). 

The combined *NPM1*/*DNMT3A* mutations were statistically associated only with a higher level of WBC count (>10,000 cells/mm^3^, *p* < 0.001, *p*_FDR_ ≤ 0.009). 

### 3.4. MCM7 SNPs rs2070215, rs1527423, and rs1534309, Somatic Mutations (FLT3, NPM1, DNMT3A), and Overall Survival of AML Patients

The investigated *MCM7* SNPs had no significant effect on OS in either univariable Cox regression analysis or multivariable regression analysis. There were significant associations between OS of AML patients and age at diagnosis > 65 years (*p* < 0.001), WBC count ≥ 10,000 cells/mm^3^ (*p* = 0.025), LDH ≥ 600IU/L (*p* = 0.003), ECOG performance status grade ≥2 at diagnosis (*p* < 0.001), ELN high- and intermediate-risk (*p* < 0.001), and presence of *FLT3* ITD mutation (*p* < 0.001), as we previously reported [30,31]. In addition, we observed an association between lower OS and combined *FLT3* ITD/D835 (*p* = 0.027) and *FLT3/DNMT3A* (*p* = 0.039) mutations.

### 3.5. Aditional Analysis

We also investigated if the presence of *FLT3* somatic mutations modified the effect of LDH ≥ 600IU/L and WBC count ≥ 10,000 cells/mm^3^ on the hazard of death in AML patients. An unadjusted analysis of interaction between LDH, WBC, and *FLT3* mutations was performed for study changes in the hazard of death, these models including only *FLT3*, LDH, or WBC as independent variables and the interaction terms. LDH level ≥600 IU/L had a significant positive effect on the hazard of death (*p* = 0.004, HR = 1.49, 95% CI: 1.13–1.95). We showed that hazard of death for patients with *FLT3* gene mutation and LDH level ≥ 600 IU/L was about two times higher than in patients without *FLT3* gene mutation with LDH level < 600 IU/L (HR = 1.80, 95% CI: 1.22–2.66), while an increased hazard of death was also observed for patients with LDH level ≥ 600 IU/L within *FLT3* strata (patients with no *FLT3* mutation: HR = 1.50, 95% CI: 1.10–2.03; patients carrying *FLT3* mutation: HR = 1.15, 95% CI: 0.60–2.20). The multiplicative interaction between LDH and *FLT3* mutations, measured on a relative scale, was HR_interaction_ = 0.80, 95% CI: 0.39–1.62, so although we noticed that the estimated joint effect of LDH level ≥ 600 IU/L with the presence of *FLT3* mutations was lower than estimated effect of LDH level ≥ 600 IU/L without *FLT3* mutations, the interaction effect was not statistically significant (*p* = 0.527).

Although WBC count ≥ 10,000 cells/mm^3^ had a significant effect on the poor OS at the univariable level (*p* = 0.026, HR = 1.35, 95% CI: 1.04–1.76) as well as *FLT3* (*p* = 0.035, HR = 1.40, 95% CI: 1.02–1.91), the interaction effect of WBC and *FLT3* was not statistically significant (*p* = 0.503, HR_interaction_ = 1.34, 95% CI: 0.57–3.14); therefore, the effect of WBC count ≥ 10,000 cells/mm^3^ had an increased hazard of death within strata of an *FLT3* mutation (WBC count ≥ 10,000 cells/mm^3^ and no *FLT3* mutations: HR = 1.22, 95% CI: 0.90–1.65; and WBC count ≥10,000 cells/mm^3^ with the presence of *FLT3* mutations: HR = 1.58, 95% CI: 0.71–3.55). 

## 4. Discussion

Previous studies suggested that *MCM7* SNPs could be used as prognostic markers for cancer [15,25,26,32]. In the present study, the association between the mentioned SNPs (rs2070215, rs1527423, rs1534309) of the *MCM7* gene and AML susceptibility were evaluated by analyzing the distribution of genotypes, alleles, and haplotypes between groups. According to our results, the variant allele (C) of the rs2070215 represented a risk factor for AML. It was reported that the variant allele of rs2070215 increases the expression of *MCM7*, therefore leading to cell proliferation, increasing the relapse rate and influencing cancer prognosis [25,26]. Our results seem to be similar to those reported in the literature regarding the risk of cancer for rs2070215 [21], but show no association between investigated *MCM7* SNPs and AML prognosis, OS, relapse rate, or somatic mutations. The frequency of rs2070215, rs1527423, and rs1534309 observed in our study was similar to that reported by the Ensembl genome browser. Our findings, regarding the relapse frequency or OS, therefore partially contradict those reported earlier by Lee et al. [15]. One explanation for the different results is the number of investigated patients (we had a threefold larger AML group), and another is the ethnic origin (European as opposed to Asian). 

However, in haplotype analysis, we noticed two opposite associations between AML risk and haplotype 1 (GAT, negative association, being a protective factor) and haplotype 6 (CAT, positive association, being a risk factor). The C and G alleles of the rs1534309 are the two different alleles of the mentioned haplotypes (GAT and CAT), suggesting the dual role of rs1534309.

None of the investigated SNPs were associated with OS, and according to our results, only the variant allele of rs2070215 seems to be important for AML susceptibility.

Regarding the association between *MCM7* SNPs, somatic mutations, and clinical features of AML patients, none of the investigated SNPs were associated with the clinical features of AML patients or with *FLT3, DNMT3A,* and *NPM1* mutations. 

Furthermore, for the AML group, considered representative for our region, we found an association between the somatic mutations and high WBC count, high blast percentage, and high LDH level, as previously reported [12,33]. Therefore, patients with a high level of LDH, blast percentage, and WBC count need not only a faster investigation for *FLT3* and *NPM1* mutations, but also to be correctly classified and receive the benefit of personalized treatment (the goal of precision medicine) in the shortest time [34,35,36,37,38]. 

In the present study, associations between somatic mutations, WBC count ≥ 10,000 cells/mm^3^, LDH ≥600 IU/L, and poor OS at the univariable level were significant. At the same time, *FLT3* mutations were associated with high WBC count (≥10,000 cells/mm^3^) and LDH level (≥600 IU/L). Moreover, we observed that only the association between high LDH level (>600 IU/L) and poor OS remained significant, irrespective of *FLT3* mutation status. Our findings suggest that LDH level ≥600 IU/L could be a useful biomarker for OS. Our observation is in line with the data reported by our group in a previous study [30], as well as with studies reported by Walaa et al. [13] and Hu et al. [14].

To the best of our knowledge, this is the first study to analyze the role of *MCM7* SNPs in the development of AML in Caucasian patients and the second to investigate the mentioned SNPs in AML patients, but on a larger AML sample group than previously reported [15]. Furthermore, this is the first study to investigate the association between *MCM7* SNPs and somatic mutations, combined somatic mutations, clinical features, and OS on a relatively large number of cases. In addition, the association between somatic mutations, clinical features, and OS were evaluated. One limitation of our study is the lack of investigation of *MCM7* gene expression and Mcm7 protein level. 

## 5. Conclusions

Our study indicates that variant genotypes of investigated *MCM7* SNPs are not associated with the risk of AML, clinical features of AML patients, prognosis, or with *FLT3*, *NPM1*, and *DNMT3A* mutations. However, the variant allele of rs2070215, in allelic model, and CAT haplotype, were associated with AML susceptibility. The investigated somatic mutations were associated with high LDH level, blast percentage, and WBC count. From the clinical and biological features of AML (age at diagnosis over 65 years, ELN high-risk and intermediate-risk, presence of *FLT3* and *DNMT3A* mutations, ECOG performance status ≥ 2, WBC count ≥ 10,000 cells/mm^3^, LDH level ≥ 600 IU/L, associated with a poor OS), only LDH level ≥ 600 IU/L was associated with an increased hazard of death, and this association remained significant when quantifying for effect modification by *FLT3* (ITD, D835) mutation status. The association of high LDH level (≥600 IU/L) and WBC count (≥10,000 cells/mm^3^) with poor OS showed the same magnitude in patients with or without FLT3 mutation. As a result of the mentioned associations between LDH level (≥600 IU/L), WBC count (≥10,000 cells/mm^3^), and presence of *FLT3* and *NPM1* mutations, as well as their impact on OS, AML patients with high LDH level and WBC count need to be promptly investigated for *FLT3* and *NPM1* mutations. 

## Figures and Tables

**Table 1:** Genotypes and alleles distribution.

Studied Genetic Models (Additive, Dominant, Allelic)	AML Patients *n* (%)	Controls *n* (%)	Crude OR ^(a)^ (95% CI: Lower Limit to Upper Limit)	*p* ^(a)^	*p*_BH_^(b)^
*MCM7* rs1534309					
CC	13 (4.6)	12 (3)	Reference	0.247	0.257
GC	99 (35.2)	127 (31.4)	0.72 (0.32–1.65)		
GG	169 (60.1)	266 (65.7)	0.59 (0.26–1.32)		
GC + GG	268 (95.4)	393 (97)	0.63 (0.28–0.283)	0.257	0.257
C allele	125 (22.2)	151 (18.6)	Reference		
G allele	437 (77.8)	659 (81.4)	0.80 (0.61–1.05)	0.102	0.257
*MCM7* rs1527423					
GG	76 (27)	88 (21.7)	Reference	0.238	0.238
AG	140 (49.8)	209 (51.6)	0.78 (0.53–1.13)		
AA	65 (23.1)	108 (26.7)	0.70 (0.45–1.08)		
AG + AA	205 (73)	317 (78.3)	0.75 (0.53–1.07)	0.109	0.164
G allele	292 (52)	385 (47.5)	Reference		
A allele	270 (48)	425 (52.5)	0.84 (0.68–1.04)	0.107	0.164
*MCM7* rs2070215					
TT	157 (55.9)	226 (55.8)	Reference	0.993	1.00
CT	104 (37)	151 (37.3)	0.99 (0.72–1.37)		
CC	20 (7.1)	28 (6.9)	1.03 (0.56–1.89)		
CT + CC	124 (44.1)	179 (44.2)	0.99 (0.73–1.36)	0.986	1.00
T allele	418 (68)	603 (74.4)	Reference		
C allele	197 (32)	207 (25.6)	1.37 (1.09–1.73)	0.007 **	0.021 *

Note. * *p* < 0.05; ** *p* < 0.01; ^(a)^ obtained from unadjusted regression analysis; ^(b)^ *p*-values adjusted for multiple comparisons using Benjamini–Hochberg procedure (*n* = 3 inheritance models); statistically significant results obtained if *p* < 0.05.

**Table 2:** Haplotype results.

Haplo-Type No.	Estimated Haplotypes rs1534309/rs1527423/rs2070215	Relative Frequencies in Controls	Relative Frequencies in AML Patients	*p* ^(a)^	Crude OR	95% CI	Adjusted OR ^(b)^	95% CI
*1*	GAT	0.28	0.23	0.028 *	0.53	0.72–0.99	0.71	0.52–0.97
*2*	GAC	0.24	0.23	0.834	0.91	0.67–1.22	0.89	0.66–1.20
*3*	GGC	0.012	0.015	0.631	1.03	0.35–1.03	0.99	0.34–2.92
*4*	GGT	0.28	0.31	0.484	Ref.		Ref.	
*5*	CGT	0.18	0.20	0.258	1.03	0.74–1.43	1.02	0.71–1.36
*6*	CAT	0.002	0.017	0.012 *	9.53	1.13–80.32	9.55	1.13–80.40
*7*	GAC	0.00	0.003	NC	NC	NC	NC	NC
*8*	CGC		0.0031	NC	NC	NC	NC	NC

Note. ^(a)^ *p*-values obtained from Score test; ^(b)^ OR adjusted for age and gender; Ref = Reference; NC = not calculated because of their rarity (frequency < 0.004 in controls and AML patients); * *p* < 0.05.

**Table 3:** Demographic and clinical patients’ features according to the genotypes for rs1534309, rs1527423, and rs2070215.

	*MCM7* rs1534309 CC	*MCM7* rs1534309 GC + GG	*p* ^(a)^	*MCM7* rs1527423 GG	*MCM7* rs1527423 AG + AA	*p* ^(a)^	*MCM7* rs2070215 TT	*MCM7* rs2070215 CT + CC	*p* ^(a)^
Age (years)									
<65 years	8 (4.3)	177 (95.7)	0.205	52 (28.1)	133 (71.9)	0.671	100 (54.1)	85 (45.9)	0.448
≥65 years	5 (5.2)	91 (94.8)		24 (25)	72 (75)		57 (59.4)	39 (40.6)	
Gender									
Woman	7 (5.3)	125 (94.7)	0.611	43 (32.6)	89 (67.4)	0.05	83 (62.9)	49 (37.1)	0.026 *
Man	6 (4)	143 (96)		33 (22.1)	116 (77.9)		74 (49.7)	75 (50.3)	
WBC (cells/mm^3^)									
<10,000	4 (2.9)	135 (97.1)	0.167	40 (28.8)	99 (71.2)	0.518	80 (57.6)	59 (42.4)	0.574
≥10,000	9 (6.3)	133 (93.7)		36 (25.4)	106 (74.6)		77 (54.2)	65 (45.8)	
PLT (cells/mm^3^)									
<40,000	5 (3.8)	125 (96.2)	0.563	33 (25.4)	97 (74.6)	0.561	72 (55.4)	58 (44.6)	0.879
≥40,000	8 (13)	143 (94.7)		43 (28.5)	108 (71.5)		85 (56.3)	66 (43.7)	
Hgb (g/dL)									
<10	2 (2.7)	72 (97.3)	0.524	19 (25.7)	55 (74.3)	0.757	42 (56.8)	32 (43.2)	0.858
≥10	11 (5.3)	196 (94.7)		57 (27.5)	150 (72.5)		115 (55.6)	92 (44.4)	
LDH level (IU/L)									
<600	5 (4.4)	108 (95.6)	0.895	34 (30.1)	79 (69.9)	0.346	66 (58.4)	47 (41.6)	0.483
≥600	8 (4.8)	160 (95.2)		42 (25)	126 (75)		91 (54.2)	77 (45.8)	
Blast (in bone marrow, %)									
<70	7 (3.9)	172 (96.1)	0.557	49 (27.4)	130 (72.6)	0.870	100 (55.9)	79 (44.1)	0.998
≥70	6 (5.9)	96 (94.1)		27 (26.5)	75 (73.5)		57 (55.9)	45 (44.1)	
AML subtype									
De novo	11 (4.8)	217 (95.2)	1	60 (26.3)	168 (73.7)	0.678	127 (55.7)	101 (44.3)	0.592
Secondary AML	2 (4.2)	46 (95.8)		14 (29.2)	34 (70.8)		26 (54.2)	22 (45.8)	
Therapy related AML	0 (0) 1	5 (100) 11		2 (40)	3 (60)		4 (80)	1 (20)	
Response									
Complete remission	2 (4.7)	41 (95.3)	0.799	12 (27.9)	31 (72.1)	0.699	25 (58.1)	18 (41.9)	0.184
Partial remission	1 (2)	48 (98)		16 (32.7)	33 (67.3)		23 (46.9)	26 (53.1)	
Resistance	3 (4.2)	68 (95.8)		18 (25.4)	53 (74.6)		35 (49.3)	36 (50.7)	
No response/Induction death	5 (7.2)	64 (92.8)		15 (21.7)	54 (78.3)		46 (66.7)	23 (33.3)	
Relapse	2 (4.1)	47 (95.9)		15 (30.6)	34 (69.4)		28 (57.1)	21 (42.9)	
ELN 2017 RISK									
Low risk	3 (5.5)	52 (94.5)	0.120	18 (32.7)	37 (67.3)	0.061	34 (61.8)	21 (38.2)	0.270
Intermediate	5 (3.4)	141 (96.6)		40 (27.4)	106 (72.6)		84 (57.5)	62 (42.5)	
High risk	3 (4.2)	68 (95.8)		13 (18.3)	58 (81.7)		33 (46.5)	38 (53.5)	
NA (Not available)	2 (22.2)	7 (77.8)		5 (55.6)	4 (44.4)		6 (66.7)	3 (33.3)	
*FLT3* ITD−	10 (4.3)	220 (95.7)	0.711	64 (27.8)	166 (72.2)	0.532	129 (56.1)	101 (43.9)	0.877
*FLT3* ITD+	3 (5.9)	48 (94.1)		12 (23.5)	39 (76.5)		28 (54.9)	23 (45.1)	
*FLT3* D835−	12 (4.5)	254 (95.5)	0.518	70 (26.3)	196 (73.7)	0.245	148 (55.6)	118(44.4)	0.741
*FLT3* D835+	1 (6.7)	14 (93.3)		6 (40)	9 (60)		9 (60)	6 (40)	
*FLT3* (ITD + D835)−	10 (4.5)	211 (95.5)	1	61 (27.6)	160 (72.4)	0.687	124 (56.1)	97 (43.9)	0.878
*FLT3* (ITD + D835)+	3 (5)	57 (95)		15 (25)	45 (75)		33 (55)	27 (45)	
*NPM1*−	9 (3.9)	219 (96.1)	0.276	60 (26.3)	168 (73.7)	0.567	123 (53.9)	105 (46.1)	0.178
*NPM1*+	4 (7.5)	49 (92.5)		16 (30.2)	37 (69.8)		34 (64.2)	19 (35.8)	
*DNMT3A*−	11 (4.5)	236 (95.5)	0.662	65 (26.3)	182 (73.7)	0.457	135 (54.7)	112 (45.3)	0.269
*DNMT3A*+	2 (5.9)	32 (94.1)		11 (32.4)	23 (67.6)		22 (64.7)	12 (35.3)	
Treatment									
HD	3 (23.1%)	140 (52.2%)	0.090	33 (43.4%)	110 (53.7%)	0.300	71 (45.2%)	72 (58.1%)	0.087
HD + HSCT	1 (7.69%)	15 (5.60%)		5 (6.58%)	11 (5.37%)		11 (7.01%)	5 (4.03%)	
LD	9 (69.2%)	113(42.2%)		38 (50.0%)	84 (41.0%)		75 (47.8%)	47 (37.9%)	
Toxicity									
Yes	6 (46.2%)	154 (57.5%)	0.605	41 (53.9%)	119 (58.0%)	0.630	92 (58.6%)	68 (54.8%)	0.610
No	7 (53.8%)	114 (42.5%)		35 (46.1%)	86 (42.0%)		65 (41.4%)	56 (45.2%)	

Note. ^(a)^ estimated *p*-values obtained from Chi-square or exact Fisher’s tests; * *p* < 0.05; bold values denoted borderline *p*-values; H = high dose, HSCT = hematopoietic stem cell transplantation, LD = low dose.
